# Combination drug strategies for biofilm eradication using synthetic and natural agents in KAPE pathogens

**DOI:** 10.3389/fcimb.2023.1155699

**Published:** 2023-04-17

**Authors:** Anurag Kumar Bari, Tanvi Sandeep Belalekar, Aruna Poojary, Seema Rohra

**Affiliations:** Department of Pathology & Microbiology, Breach Candy Hospital Trust, Mumbai, India

**Keywords:** antimicrobial resistance (AMR), combination therapy, biofilms, quorum sensing _(QS)_, KAPE pathogens

## Abstract

Antibiotic resistance is a global threat caused by factors such as overuse of antibiotics, lack of awareness, development of biofilms etc. World Health Organization released a list of global priority pathogens which consisted of 12 species of bacteria categorized as expressing critical, high and medium resistance. Several Gram-negative and Gram-positive species are known to cause wide varieties of infections and have become multidrug or extremely drug resistant. Pathogens causing infections associated with invasive medical devices are biofilm producers and hence their treatment becomes difficult due to a structurally stable matrix which prevents antibiotics from penetrating the biofilm and thereby showing its effects. Factors contributing to tolerance are inhibition of penetration, restricted growth and activation of biofilm genes. Combination drug therapies has also shown potential to eradicate biofilm infections. A combination of inhaled Fosfomycin/tobramycin antibiotic strategy has been effective against Gram-negative as well as Gram positive organisms. Along with antibiotics, use of natural or synthetic adjuvants shows promising effects to treat biofilm infections. Fluroquinolone activity on biofilms is disrupted by low oxygen tension in the matrix, a strategy known as hyperbaric oxygen treatment that can enhance efficacy of antibiotics if well optimized. Adjuvants such as Ethylenediaminetetraacetic acid (EDTA), Sodium Dodecyl Sulphate (SDS) and chlorhexidine act by killing non-growing microbial cells aggregated on the inner layer of the biofilm. This review aims to list down current combination therapies used against Gram-negative and Gram-positive biofilm forming pathogens and brief about comparison of combination drugs and their efficacies.

## 1 Introduction

Bacterial biofilms cause infections which when left untreated cause increase in antimicrobial resistance in the environment. Currently, resistance has developed towards Beta-Lactams and other antimicrobials that are commonly used to cure bacterial diseases. According to a paper published in the Lancet, antimicrobial resistance poses a serious threat to global public health, killing at least 1.27 million people worldwide and being linked to over 5 million deaths in 2019 (Antimicrobial Resistance Collaborators, 2022). The inappropriate use of antibiotics is responsible for antibiotic pressure, making them less effective leading to the emergence of “superbugs”. Furthermore, the protective extracellular polymeric material matrix restricts the drugs’ ability to diffuse throughout the biofilm. The barriers of the Gram-negative bacterial membrane and the biofilm matrix are believed to be permeable to polymers when chemical groups on the polymer scaffolds are carefully engineered. These polymeric nanoparticles boost antibiotic uptake by Gram-negative bacteria and the biofilm matrix, boosting the effectiveness of antibiotics used in combination therapy (Gupta et al., 2020).

Factors that govern biofilm formation are i) systems of communication between cells, such as quorum sensing (QS), in which the production of signaling molecules by the cells controls the formation of the biofilm, ii) bacterial secondary messengers that regulate flagellar attachment that leads to attachment on the surfaces and iii) EPS Matrix components. Both manmade and natural environments are capable of supporting biofilms. Expression of certain genes triggers formation of EPS matrix; *treC* and *sugE* are the genes responsible for capsular polysaccharide production in *K.pneumonieae* (Wu et al., 2011). It is well known that the presence of biofilm-related genes in *Acinetobacter* sp., such as *bap*, *bla_PER-1_*, *csu*E, and *omp*A, leads to the creation of biofilms and antimicrobial resistance (Yang et al., 2019). Similar gene *Mva*U in *P.aeruginosa* is responsible for the *Mva*T-specific regulation of *cup*A genes. As a result, this gene seems to be a significant regulatory element within a complicated network that regulates the development and maturity of biofilms in *P.aeruginosa* (Vallet et al., 2004). In E. coli, biofilm genes are categorized based on functionality such as motility (*flg*, *flh*, *fli* and *mot*), Type 1(*fim* A, B, C, D, F, G, H), curli formation (*csg*) and LPS formation (*lpc*, *gmh*, and *rfa*) along with other genes (Niba et al., 2007). Treatment options include, removal of the invasive device once it is not necessary, individual antibiotic therapy and combinations that are currently being used. Unfortunately, removing the device is not always possible and hence drug therapy that promotes biofilm destruction needs to be studied. Drug targets in biofilm mediated infections are extra polymeric substance matrix highly composed of protein and carbohydrate materials that are stable and difficult to penetrate. Along with EPS, planktonic bacterial cell aggregate’s Quorum sensing pathways can also be potential drug target for eradication of biofilms.

## 2 Manuscript formatting

### 2.1 Stages of Biofilm formation

**Figure 1:**
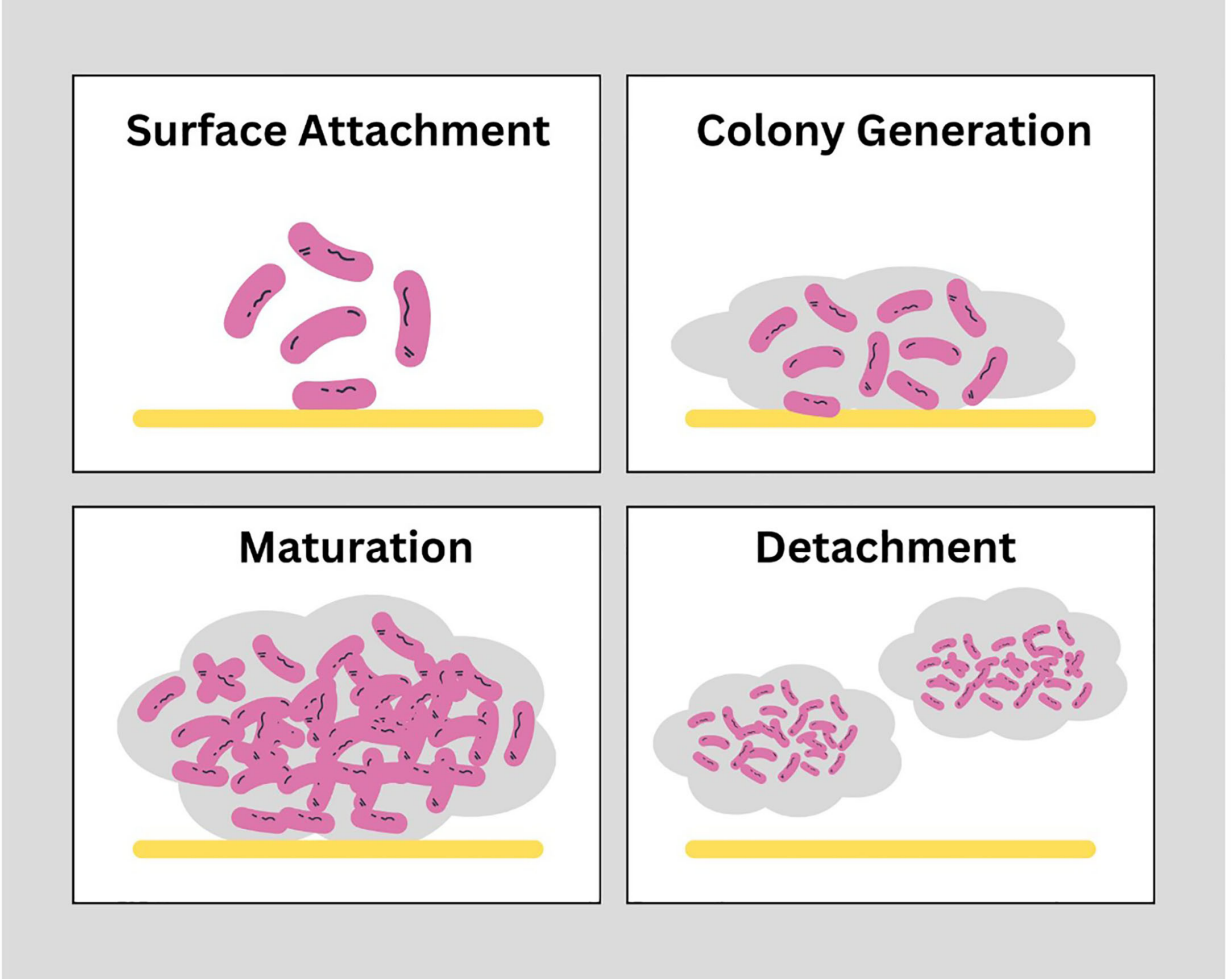
Stages of biofilm formation: Biofilms are structurally strong which are developed in 4 stages. Surface attachment: Involves reversible interaction of proteins of free-swimming planktonic bacterial cells with the surface of host cells or medical devices to achieve stability. Colony generation: Microcolonies within the matrix of biofilm replicate and double in number. Maturation: Complex structure of EPS matrix is formed with higher bacterial load within the structure. Detachment: The final stage is dispersion of cells from matrix into outer environment. (https://www.canva.com/).

Biofilms are composed bacterial cell aggregates and extra polymeric substances. These structures when formed on medical devices used for treatment purposes are hard to remove with physical and chemical shear forces. In general, there are 4 major stages of biofilm formation shown in **Figure 1**. a) Bacterial attachment to a surface: small number of bacterial cells when transported into the system or on the medical device, with the help of flagellum and adherence proteins adheres to the surface and initiate a colony. b) Colony formation: After attachment, uptake of nutrients from host cell leads to bacterial colony development. c) Maturation of biofilm: When bacteria multiply, adhesion mechanisms hold them together and disruptive activities create channels in the biofilm structure are part of the biofilm maturation process and d) Detachment: After maturation, dispersion of cells from biofilm in surroundings takes place in the last stage. The phenotype of bacterial cells within the biofilm is different than in its planktonic stage. Biofilms provides some additional protection from environmental destruction to the cells present within the biofilms and is also impermeable to antibiotics.

### 2.2 Biofilm mediated infections

The first pathogen on the critical priority list of diseases for innovative antibiotics to turn into a “red-alert” human pathogen belongs to the *Acinetobacter* species, specifically Carbapenem Resistant *Acinetobacter baumannii* (CRAB). The most common types of biofilm-associated infections caused by *A. baumannii* are catheter-related blood stream infections and ventilator-associated pneumonia Gedefie et al., 2021).

Another major hazard to patients is frequently posed by *K.pneumonieae* due to the rapid emergence of multidrug resistant (MDR) & extensively drug resistant (XDR) strains and the associated high mortality rate due to the diminished efficacy of available therapeutic choices (Nirwati et al., 2019). The transmission of antibiotic resistance genes from environmental bacteria to clinically significant bacteria is another crucial problem encountered with *K. pneumonieae*. It has an important role in causing opportunistic infections that affect people with immunosuppressed conditions such diabetic mellitus, chronic obstructive lung disease (COPD) and chronic kidney disease (CKD) to name a few. Virulence factors are utilized by *K. pneumonieae* both during biofilm formation and for survival while evading the immune system. It has the ability to form a thick layer of extracellular biofilm that maintains the bacterial attachment to living or inactive surfaces, preventing antibiotic penetration and reducing its effects (Nirwati et al, 2019).

One of the main causes of nosocomial infections, which afflicts over 2 million people annually and results in about 90,000 fatalities, is *Pseudomonas aeruginosa*, a widespread microorganism (Mulcahy et al., 2014). Ability of *P. aeruginosa* to form a biofilm, improves its capacity to cause infections by shielding them from host defenses and chemotherapeutic agents. The potential of *P. aeruginosa* to develop biofilms on implanted and indwelling devices has long been an issue in patient care (Mulcahy et al., 2014).

Similar to other critical pathogens *Enterobacter* species which are commonly occurring pathogens including *Enterobacter cloacae*, *Enterobacter aerogenes* and other *Enterobacter* species are also known to form biofilms which mediate resistance. Uropathogenic *E. coli* that forms biofilms is linked to chronic and persistent inflammation, which can cause severe or recurrent urinary tract infections (Niranjan and Malini, 2014). Biofilms provide conditions where antibiotics are poorly absorbed and virulence genes are transferred horizontally, favoring the growth of multidrug-resistant organisms (MDRO).

### 2.3 Combination antimicrobial therapies on bacterial biofilms

Combination therapy against biofilms includes combinations of two or three chemically different candidates such as antimicrobial agents along with natural and synthetically prepared molecules to obtain a synergistic effect. Biofilms are difficult to destroy with a single agent hence, recent research is focused on developing combinatorial drugs to either enhance penetration of existing antimicrobials into the rigid biofilm structure or directly eradicate the strong biofilm matrix. Some of the studies also included anti-cancer agents which had proven to be effective against biofilms. Yuan et al., studied the repurposing of anti-cancer agent Cisplatin which was found effective in treating *P. aeruginosa* mediated biofilms infection. It was successful in eradicating biofilms in a murine keratitis model (Yuan et al., 2018). Another anti-cancer agent an uracil analog, 5-Fluorouracil, used in treatment of various cancers such as pancreatic, colorectal and some skin-cancers was also tested against *E. coli* strains and was found to be effective in decreasing biofilm formation in dose dependent manner and also repressed virulence genes in the same (Attila et al., 2008).

In this review, twenty combination therapies are discussed summarized in **Table 1** that have recently been studied and found to be successful in eradicating the bacterial biofilms. The study conducted by Aiyer et al. in 2021 on patients of Cystic fibrosis. Cystic fibrosis is a genetic disorder affecting the ciliated mucosal surfaces of the body. A mutation in Cystic Fibrosis Transmembrane Conductance Regulator (CFTR) gene leads to imbalance of chlorine and bicarbonate ions in the body. This imbalance results in generation of thick, static mucous especially in the lungs which further gets colonized by the bacteria and produces a thick biofilm. This study showed that a combination of N-Acetylcysteine NAC (4890ug/ml) and Ciprofloxacin (32 or 64ug/ml) had a synergistic effect. This combination also showed antibiofilm activity against *Pseudomonas aeruginosa* and other microbes, NAC was thought to inhibit EPS matrix production which is one of the significant steps in destroying the rigidity of the biofilm (Aiyer et al., 2021). The study conducted in 2014 reviewed biofilm mediated urinary tract infections. Biofilms formed in uroepithelium cells can lead to a serious infection in the kidneys i.e pyelonephritis. According to the review, Macrolides were the first choice to use as a combination antibiotic, Clarithromycin along with vancomycin was found to destroy the biofilm forming bacterial cells as well as the planktonic cells which can be effective in destroying the biofilm completely thereby resolving the infection. This combination is active against Gram negative bacteria and specifically proven effective against *Pseudomonas aeruginosa* and *Staphylococcus* sp. This combination targets the major component of the EPS matrix i.e., the alginate which is thick and solid difficult to destroy preventing the entry of antibiotics. Another combination of a macrolide and a carbapenem, i.e roxithromycin and imipenem helps white blood cells penetrate inside the matrix and destabilize the biofilm eventually eradicating it (Soto, 2014). Another study conducted using a murine model on chronic respiratory infections demonstrated interference of biofilms formed by *Pseudomonas aeruginosa*. This study used a combination of clarithromycin and levofloxacin with an efficacy rate of 99% that was effective against the bacterium compared to individual antibiotic therapy (Yanagihara et al., 2000). The next combination therapy that has been tested is a combination of an antibiotic and a chelating agent i.e., colistin and EDTA to overcome the biofilms formed on medical devices such as vascular catheters by colistin resistant *Klebsiella pneumonieae*. XDR pathogens were chosen in this recent study and it was concluded that combination of colistin and EDTA was successful, both in planktonic and cells forming biofilms with an efficacy ranging between 90-100%. The concentrations of antimicrobials used in the study were 0.25 to 1ug/ml for colistin and 12mg/ml of EDTA which are relatively low, preventing damage to other normal body cells (Shein et al., 2021). Biofilms formed on dental surfaces are mainly due to two important bacteria *P. gingivalis* and *S. mutans*. Triclosan’s antibacterial activity was enhanced by oligoG, especially when combined at 0.3% against S. mutans cultured in artificial saliva. When fighting against established *P. gingivalis* biofilms, OligoG did not perform optimally indicating the combination to be synergistic. This study was carried out by Jessica Louise Roberts et al. in 2013. Combination of OligoG and Triclosan was effective in treating the biofilms caused by the bacteria (Roberts et al., 2013).

**Table 1:** Summarization of combination therapies against gram-negative and gram-positive organisms (N/A- Not Available).

Sr. Non	Antibiotic Combination	Tested Combination	Efficacy rate	References
Natural-Antibiotic Combination				
**1**	NAC and Ciprofloxacin	*P.aeruginosa* and other CF pathogens	N/A	Aiyer et al., 2021
**2**	OligoG and Triclosan	*S. mutans* and *P.gingivalis*	N/A	Roberts et al., 2013
**3**	Tobramycin and G10KHc (Peptide), DJK-5 and DJK-6 and Ciprofloxacin, ceftazidime and tobramycin	*P. aeruginosa, A. baumannii, K.pneumoniae, E. coli, P.aeruginosa*	99.99%, 98-100%	Grassi et al., 2017
**4**	Minocycline and EDTA, Linezolid and Heparin, Vancomycin and Heparin, Cotrimoxazole and Heparin	Gram-negative organisms	N/A	Ciofu et al., 2017
**5**	Polymers and Colistin	Gram-negative	N/A	Gupta et al., 2020
**6**	Light Stimuli Responsive Therapy	*E.coli, E.cloacae, S.aureus* and MRSA	99-100%	Sikder et al., 2021
**7**	Curcumin and Antimicrobials	Gram negative and Gram Positive	57%	Kali et al., 2016
**8**	Polymixin B and Gramicidin S	MDR *P.aeruginosa*	71%	Berditsch et al., 2015
**9**	Nisin and Polymixins	*P.aeruginosa*	100%	Field et al., 2016
**10**	Terpenes and Bacterial Antibiotics	Gram-negative and Gram-positive	40-91%	Zacchino et al., 2017
Antibiotic-Antibiotic Combination				
**11**	Clarithromycin and Vancomycin, Roxithromycin and Imipenem	Gram-negative spp	N/A	Soto, 2014
**12**	Clarithromycin and Levofloxacin	*P.aeruginosa*	99.9%	Yanagihara et al., 2000
**13**	Colistin and EDTA	*K.pneumonieae*	90-100%	Shein et al., 2021
**14**	Melitin and Colistin with other antimicrobials	*P.aeruginosa, K. pneumonieae and E.coli*	20-40%	Dosler et al., 2016
**15**	Daptomycin with other antimicrobials	*S.aureus*	N/A	Gould et al., 2013
**16**	Fosfomycin and Colistin	*E.coli, K. pneumonieae, P.aeruginosa and A.baumanni*	10% Synergism	Boncompagni et al., 2022
**17**	Prulifloxacin and Fosfomycin	*P.aeruginosa*	90%	Mikuniya et al., 2007
**18**	FmOC and Phenylalanine	*S.aureus* and *P. aeruginosa*	50% ECM components degradation	Singh et al., 2021
**19**	Baicalin Hydrate and Tobramycin, ACNQ and Ciprofloxacin	*P.aeruginosa*	68-90%	Hawas et al., 2022
**20**	CarboxyTEMPO and Ciprofloxacin	*P.aeruginosa and E.coli*	90-99%	Reffuveille et al., 2015

A review by Lucia Grassi et al. in 2017 gives a brief account of use of various antimicrobial peptides in combination with antibiotics in treating biofilm mediated infections caused by *Klebsiella* sp., *Pseudomonas aeruginosa*, *Enterobacter* sp. etc. From the review, 2 major combinations were G10KHc along with tobramycin which is effective against pseudomonal biofilms and DJK5 and DJK6 with tobramycin, ciprofloxacin and ceftazidime against wide range of Gram-negative organisms. The combinations were found to be 98-100% efficient (Grassi et al., 2017). A review published in 2017 gives a brief understanding of the underlying mechanisms of development of biofilms and listed down some of the effective combined antibiotics to treat biofilms. Biofilms formed on medical devices such as catheters can be treated by combining Minocycline(3mg/ml) and EDTA (30mg/ml), Linezolid(2mg/ml) and Heparin(2000U/ml), Vancomycin(2.5mg/ml) and Heparin(2500U/ml) and Cotrimoxazole(10mg/ml) with Heparin(2500U/ml) (Ciofu et al., 2017). A recent review published in frontiers in 2022, has given an account of combination therapies against biofilms formed by *Pseudomonas aeruginosa*. Two combinations, one of which is Baicalin Hydrate along with tobramycin is categorized as quorum sensing inhibitor and it acts on pseudomonal biofilms with an efficacy rate of 60-90% and another combination of 3-amino-7-chloro-2-nonylquinazolin-4(3H)-one (ACNQ) with ciprofloxacin shows an efficacy rate of 80%. This review also covers more combinational antibiofilm therapies acting against Gram-positive and Gram-negative organisms (Hawas et al., 2022). The next study is conducted on MDR Gram-negative pathogens and it covers the use of polymeric nanoparticles that can be effectively penetrated inside the biofilm and combining it with an antimicrobial agent which will enhance the activity of that antimicrobial to destroy the stability and the biofilm forming cells inside the matured biofilm structure. In this study the polymeric nanoparticles are combined with colistin and had shown a synergistic effect with a Fractional Inhibitory Concentration value ranged from 0.3-0.5 and the combination has also been successful in reducing the colistin dosage up to 16-fold which is an advantage to secure normal cells (Gupta et al., 2020). Reffuveille et al. in 2015, had successfully demonstrated eradication of biofilms formed by pseudomonas aeruginosa strain PA14 (99.3%) and *Escherichia coli* O157 (93%), using Nitroxide Carboxy-TEMPO (20uM) in combination with ciprofloxacin concentration of 320ng/ml for pseudomonal strain and 20ng/ml for *E. coli* strain (Reffuveille et al., 2015). For several resistant Gram-negative pathogens, colistin and carbapenems are the choice of drugs for treatment of the disease. In the study conducted in 2016, to treat biofilm mediated infections caused by Gram-negative pathogens, antimicrobial cationic peptides, Melittin and Colistin combinations were evaluated. The most effective combination evaluated was against pseudomonas aeruginosa i.e., colistin and ciprofloxacin. Combination of colistin and imipenem was used against *E. coli* and *Klebsiella pneumonieae*. Efficacy of the antibiotic-antibiotic combinations ranged from 20-40% (Dosler et al., 2016). The next review briefs about combination therapy against *S. aureus* which causes skin and tissue infections. The study includes combination of low dose as well as high dose daptomycin with other antimicrobials (Gould et al., 2013).

A new therapeutic approach was reviewed in an article in 2021 that describes the application of stimuli responsive therapy along with drug combinations. This experimentation made use of phototherapy, The two main techniques employed in light-induced therapy are photodynamic therapy (PDT) and photothermal therapy (PTT). Light induced eradication has a 99-100% efficiency rate. Some of the light-induced therapies are PDT and Ampicillin used against *E. Coli* K12-MG 1655 processed on carbon dot platform, PTT and Ciprofloxacin was effective against *E. coli*, *S. aureus* processed on hydrogel, and ferulic acid/sulfur dioxide against *E. cloacae* MTCC 509 (Sikder et al., 2021). An original research article by Arunava Kali et al. in 2016 studied combination of a natural entity with an antibiotic to obtain synergism. This study evaluated the interaction of curcumin and antibiotics *in vitro* against 60 isolates of bacteria that produced biofilm. Curcumin’s median inhibitory concentrations (MICs) for both Gram-positive and Gram-negative isolates were 127 mg/L and 117 mg/L, respectively. Gram-positive isolates showed the greatest synergy with ciprofloxacin, while Gram-negative isolates showed the greatest synergy with amikacin, gentamicin, and cefepime. Efficiency rate of curcumin was about 57%, curcumin being a natural component possess advantages and can be used in large amounts with minimal side effects (Kali et al., 2016). This work used a quantitative checkerboard assay using resazurin as a growth indicator to examine the interaction between two cyclic antimicrobial peptides, PMB and gramicidin S (GS), against various *P. aeruginosa* isolates. When compared to treatment with the individual peptides, the peptide combinations greatly reduced the development of planktonic bacteria. Additionally, compared to single-peptide treatments, the combination of PMB and GS had a quicker and longer lasting effect on the metabolic activity of pre-grown biofilms. The combination showed 71% efficacy. This combination can also be used as a topical medication to prevent infection caused by MDR *Pseudomonas aeruginosa* isolates (Berditsch et al., 2015). The next study investigated fosfomycin, colistin, and their combinations having *in vitro* action against planktonic and biofilm cultures of Gram-negative microorganisms by chequerboard assay. Fosfomycin and colistin demonstrated considerable synergy against MDR Gram-negative bacteria growing in biofilm at quantities obtainable by inhaling nebulized medications (Boncompagni et al., 2022). Potential antibiofilm capabilities could be found in the recently identified antibacterial Fmoc-phenylalanine (Fmoc-F) and other Fmoc-amino acids (Fmoc-AA) with surfactant qualities evaluated in a study conducted in 2020. Methods used for evaluation are crystal violet staining, scanning electron microscopy procedure, Attenuated Total Reflection - Fourier Transform Infrared Spectroscopy and some biochemical assays. This combination was effective in inhibiting *S. aureus* and Pseudomonas biofilms as it reduced ECM components (50%) directly affecting the stability of biofilm structure. Synergy is obtained when Fmoc-F is combined with vancomycin and ampicillin antibiotics (Singh et al., 2021). A study performed by Des Field et al. evaluated synergistic action of Nisin along with Polymixins for controlling infections caused by pseudomonas biofilms. Nisin is thought to increase efficacy of polymixins and the concentrations of polymixins can be effectively reduced by combining with nisin which is effective to reduce polymyxin toxicity. Colistin or polymyxin were used in investigations on biofilm prevention at concentrations of MIC (0.78, 0.31, and 0.15 g/ml, respectively), while nisin was used at MIC (50 and 5 ug/ml) (Field et al., 2016). The therapeutic effectiveness of prulifloxacin (PUFX) against *Pseudomonas aeruginosa* was the main focus of the study conducted in 2007. It combined PUFX with Fosfomycin. PUFX at a dose of 20 mg/kg and FOM at a dose of 100 mg/kg showed a distinct synergistic effect (Mikuniya et al., 2007). The study from the review in 2017 tends to list down possible combinatorial therapies against bacteria and fungi. The combination used against *E.coli* and Pseudomonas in the review included Ciprofloxacin along with Asiatic/Urosolic acid and Tobramycin along with Asiatic/Corosolic acid respectively. *S.aureus* biofilms were treated by combining Gentamicin/Nafcillin with Farnesol and Oxacillin with Salvipisone. This review was focused on listing down combination of natural potentiators with synthetic antimicrobials (Zacchino et al., 2017).

## 3 Conclusion

The stable structure of biofilms causes obstacles in therapy specifically disabling drug delivery systems and reducing the efficacy of antibiotic agents. Individual antibiotic therapies are known to become less effective on biofilm forming pathogens, hence studies on combination therapies have gained importance and proven effective in enhancing the activity of existing antibiotic or destroying the biofilms. This review intended to give a brief account on currently established combinatorial therapies using natural as well as synthetic entity along with existing antimicrobials to enhance eradication of biofilms present in host cells and medical devices. It is important to identify the genetic markers that induce biofilm production in the bacterial cell and analyze individual component of EPS matrix. Analysis should also be focused on quorum sensing mechanisms of pathogens as a potential drug target to destroy biofilms completely. Combining a synthetic entity with antibiotics has disadvantages of disrupting normal cellular functioning whereas, combination of natural agent with an antibiotic could be a promising solution as natural components have lesser side effects and can be used in large concentration with lower concentrations of synthetic antimicrobial agents.

## Author contributions

AB and TB contributed to conception and design of the study, acquisition, analysis, interpretation of data, drafting and ensuring that questions related to the accuracy or integrity of any part of the work are appropriately investigated and resolved. AP and SR contributed to manuscript revision, read, and approved the submitted version. All authors contributed to the article and approved the submitted version.
